# Comprehensive assessment of *Erwinia amylovora*: from establishment risk in global host production areas to dispersal dynamics and associated economic losses in China

**DOI:** 10.3389/fpls.2025.1641129

**Published:** 2026-02-04

**Authors:** Ming Li, Xiaoqing Xian, Zhenan Jin, Yuhan Qi, Jianyang Guo, Nianwan Yang, Guifen Zhang, Jin Xu, Wanxue Liu

**Affiliations:** 1 State Key Laboratory for Biology of Plant Diseases and Insect Pests, Key Laboratory for Prevention and Control of Invasive Alien Species of Ministry of Agriculture and Rural Affairs, Institute of Plant Protection, Chinese Academy of Agricultural Sciences, Beijing, China; 2 Western Agricultural Research Center, Chinese Academy of Agricultural Sciences, Changji, China

**Keywords:** dispersal risk, economic impact, invasive plant pathogens, overlapping area, quantitative assessment

## Abstract

*Erwinia amylovora* is the bacterial pathogen that causes fire blight and is considered one of the most important plant pathogenic bacteria in the world, posing a serious threat to pear and apple production. However, majority of the current risk assessment studies have focused primarily on the potential geographic distribution of *E. amylovora*, with less focus on its dispersal patterns, dispersal risk areas, and economic impacts. Here, species distribution models, the minimum cost arborescence approach, the MigClim package, and Monte Carlo stochastic simulations were integrated to comprehensively assess the global establishment risk, the local dispersal patterns, the dispersal risk areas, and the economic losses for *E. amylovora*. The results showed that *E. amylovora* is primarily distributed in North America, southern South America, Europe, northern and southern Africa, western and eastern Asia, and southern Oceania under near-current climatic conditions. In addition, the overlapping area between the distribution area of *E. amylovora* and the host production area is 1,897.62 × 10^4^ km^2^, mainly located in central North America, southern South America, Europe, northern Africa, eastern and western Asia, and southern Oceania. Its global distribution and the overlapping areas are expected to expand further under future climatic conditions. *Erwinia amylovora* shows a primarily “leap-frog” long-distance spread in China, and the dispersal risk area is mainly in northwestern China. The economic losses caused by *E. amylovora* to the host industry amounted to 5,603.66 million dollars without any control measures; however, 2,390.13 million dollars can be saved after control measures. Such comprehensive risk assessments provide global guidance for the monitoring and control of *E. amylovora* in host production areas while also helping to formulate management priority strategies in local dispersal risk areas, thereby reducing economic impacts.

## 1 Introduction

Invasive alien species (IAS) and climate change have emerged as escalating global threats, leading to biodiversity reduction and economic losses and posing a threat to public health (Diagne et al., 2021; Lopez et al., 2022; Zhang et al., 2022). Due to the globalization of trade and agriculture, invasive plant pathogens (IPPs) and their vectors have been introduced into new regions, posing unexpected risks to global food security (Montgomery et al., 2023). At the same time, climate change is reshaping crop–microbe interactions and exacerbating the frequency and severity of plant diseases (Singh et al., 2023). Climate warming increases the probability and spread of plant diseases, posing a substantial threat to food security (Coakley et al., 1999; Singh et al., 2023). For example, citrus huanglongbing disease has negatively affected 95% of citrus orchards in Florida, with citrus production declining by more than 60% compared to 20 years ago (Kramer et al., 2020; Wang et al., 2022). Tomato bacterial canker is a devastating disease caused by *Clavibacter michiganensis* subsp. *michiganensis*, which can reduce tomato yields by 10% and, in severe cases, by up to 80% or 100% (Zhang et al., 2012; He et al., 2016). The establishment and the dispersal of IPPs constitute critical phases in disease invasion; however, current risk assessment studies have predominantly focused on species establishment risks. The dispersal routes of IPPs are diverse and complex, and understanding their dispersal patterns and trends at the local scale enables precise interception (Essl et al., 2015; Montgomery et al., 2023; Chapman et al., 2017). Therefore, comprehensive assessments of the global distribution patterns, local dispersal patterns, dispersal trends, and the economic impacts of IPPs are needed to support effective invasion management and accurate monitoring strategies.

Fire blight, which is caused by *Erwinia amylovora*, is a highly destructive disease that affects rosaceous plants globally and particularly poses a serious risk to the pear and apple industries worldwide (Ham et al., 2022; Gong et al., 2024; Bühlmann et al., 2014; Kharadi et al., 2021). Fire blight spread after it was first reported in New York in 1780. By 1915, it had spread throughout the United States (Ham et al., 2022) and, by 1919, 1956, and 1960, to Oceania (New Zealand), Europe (United Kingdom), and Africa (Egypt), respectively (CABI, 2024). It was discovered in Xinjiang, northwestern China, in 2015 (Li et al., 2022). The disease initiates when bacterial pathogens infect the host through flower nectarthodes and through wounds, subsequently colonizing through the xylem system, rapidly infecting the entire blossom and young shoots within a few days, and then spreading to the entire plant within a few months, resulting in systemic infection (Smits et al., 2013; Borruso et al., 2017). Typical symptoms include flower necrosis, branch wilting, blackening of the surface, and woody tissue cankers, accompanied by a viscous exudate (Borruso et al., 2017; Ham et al., 2022). In 2017, fire blight affected all pear-growing regions of Korla City across approximately 6,700 hm^2^, resulting in 30%–50% yield losses and complete orchard destruction in severe cases (Huang et al., 2022). *Erwinia amylovora* is distributed in more than 60 countries and regions worldwide, including confirmed reports in 52 counties across China (EPPO, 2024). However, under climate change, the global colonization risk of *E. amylovora*, its spread patterns and trends in China, and the economic damage are still unclear, which makes accurate monitoring and control challenging.

Risk assessment is the basis for conducting IAS management by identifying uncertain events across multiple invasion stages, the aim of which is to minimize the costs of various adverse outcomes with minimal management outlay (Ding et al., 2006; Wang et al., 2007). The use of quantitative assessment models has become an important tool in the field over the last 30 years (Li and Qin, 2018), mainly for the assessment of the potential geographic distribution, the dispersal risk, and potential economic losses. Predicting the potential geographic distribution of IAS is a major component of risk assessment, and species distribution models (SDMs) are primarily constructed based on the ecological niche theory combined with specific machine algorithms (Elith and Leathwick, 2009; Fang et al., 2021). SDMs have been widely used to predict the potential geographic distribution of IPPs, and research findings can provide a theoretical basis for the prevention and control of target species (Ajene et al., 2020; Xu et al., 2020). Understanding the local dispersal patterns and dispersal risk area levels of invasive pathogens is critical for improving early warning and eradication programs (Jones et al., 2021; Zhao et al., 2024). The minimum cost spanning tree algorithm, based on the network analysis theory, was used to reconstruct the dispersal routes of IAS, which were used to estimate the dispersal patterns and distances (Hordijk and Broennimann, 2012; Cola et al., 2017). In addition, the MigClim package was used to predict the dispersal risk areas of IAS, providing a basis for prevention and control (Engler et al., 2012; Jin et al., 2024). The prediction of potential economic losses caused by IAS typically begins with an analysis of the various possible losses, the establishment of a comprehensive evaluation index system, and then simulation of the results using specific algorithms or software (Li and Qin, 2018). The @RISK software, which is based on Monte Carlo stochastic simulation methods for risk analysis, can be used to simulate possible outcomes and occurrence probabilities under any scenario using probability distributions. It has been widely applied to estimate potential economic losses caused by IAS (Cook et al., 2011; Wei et al., 2022; Xi et al., 2022).

This study used the niche concept, the minimum cost arborescence approach, the MigClim package, and Monte Carlo stochastic simulation to comprehensively assess the establishment risk of *E. amylovora* in host production areas globally, as well as to understand the local dispersal routes, the dispersal risk areas, and the economic losses caused to hosts. The main objectives were as follows: (1) to predict potential suitable areas for *E. amylovora* and identify threat areas to the host production areas globally; (2) to identify the dispersal patterns of target species in China and assess their subsequent dispersal risk areas; and (3) to quantify the economic impact of target species on the pear and apple industries under different control scenarios. The research results can provide specific control and monitoring areas for *E. amylovora* in various countries while curbing the further increase of the dispersal risk in the region.

## 2 Materials and methods

### 2.1 Global distribution records of species and environmental variables

**Figure 1:**
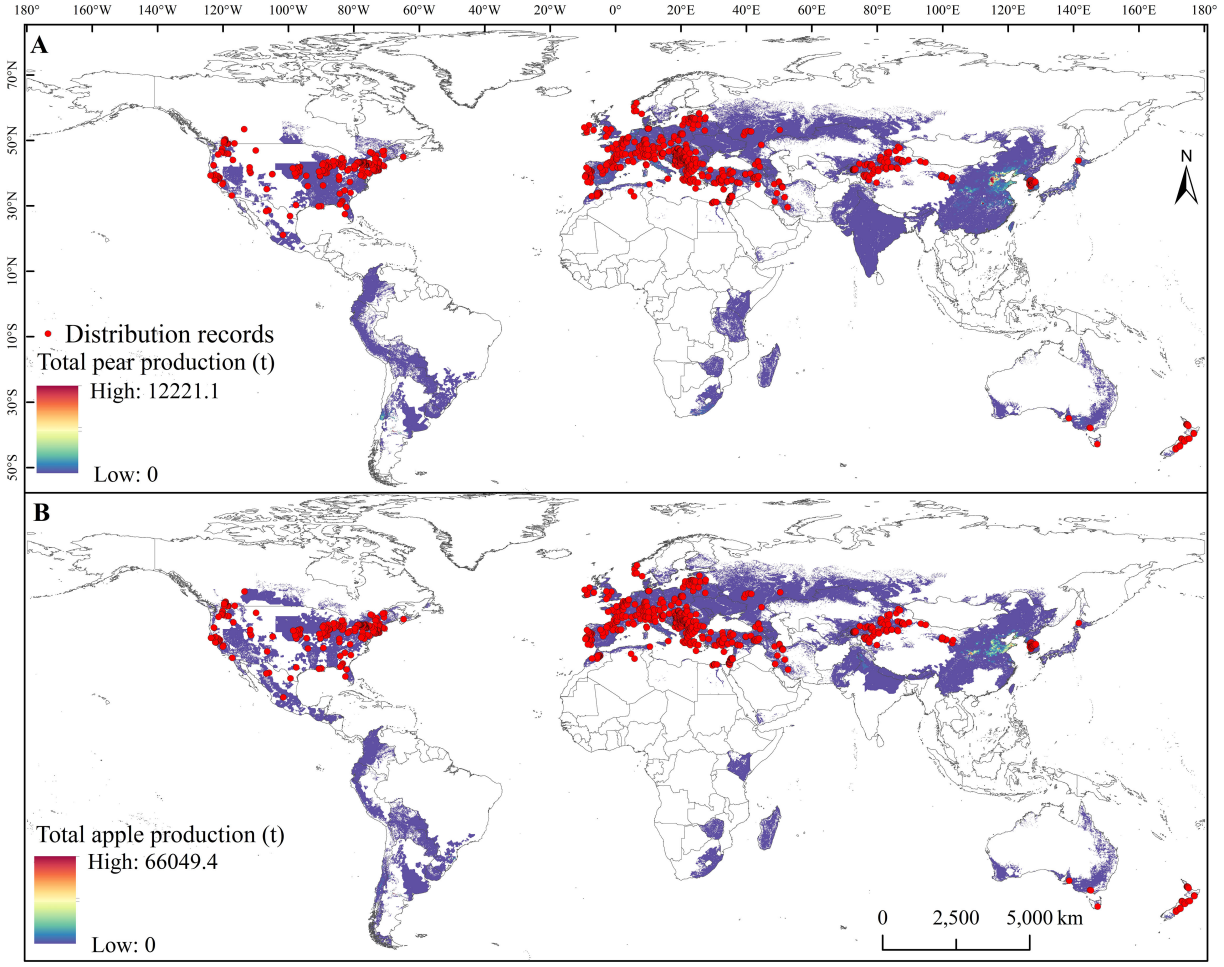
Global distribution records of *Erwinia amylovora* in total production areas within pear **(A)** and apple **(B)** grid cells. Unit: metric tons (t).

The global distribution records for *E. amylovora* were obtained from the Global Biodiversity Information Facility (GBIF.org, 2024), the EPPO Global Database (https://gd.eppo.int/), field surveys, and published literature. Distribution records with incomplete distribution information were deleted and the remaining records were standardized to decimal degrees for latitude and longitude. To avoid model overfitting caused by clustering of the species distribution records, ENMTools was used to filter the species distribution records based on the resolution of the environmental variables (Warren et al., 2010). Only one distribution record was retained within each 2.5-arcmin raster. Finally, 806 distribution records of *E. amylovora* were used for model construction (**Figure 1**).

A total of 19 bioclimatic variables were obtained from the WorldClim website (https://worldclim.org/) at a 2.5-arcmin resolution (**Supplementary Table S1**). The near-current period for bioclimatic variables was 1970–2000, and the future periods were the 2030s (2020–2040) and the 2050s (2040–2060). The future bioclimatic variables used were from the medium-resolution Beijing Climate Center Climate System Model 2 (BCC-CSM2-MR), which included three scenarios: the low forcing scenario (SSP1-2.6), the medium forcing scenario (SSP2-4.5), and the high forcing scenario (SSP5-8.5). The Human Influence Index (HII) was downloaded from the Earthdata (https://www.earthdata.nasa.gov/) at a resolution of 1 × 1 km. Altitude was obtained from the World Climate database (http://www.worldclim.org/) at a 2.5-arcmin resolution. The HII data were resampled in ArcGIS to achieve a 2.5-arcmin resolution in order to match the resolution of the other environmental variables. The presence of multicollinearity among the environmental variables can easily lead to spatial autocorrelation in the model (Ning et al., 2018). Therefore, it is necessary to screen the environmental variables. Firstly, all species distribution records and environmental variables were imported into the biomod2 platform to assess the contribution of each environmental variable to the species distribution. Subsequently, all environmental variables were imported into ArcGIS to analyze their correlations using the band collection statistical tool. If the absolute value of the correlation between two environmental variables exceeded 0.8 (|*r*| > 0.8), the environmental variable with the larger contribution was retained. The retained environmental variables were used to construct the final model (**Supplementary Figure S1**).

### 2.2 Model construction and accuracy evaluation

This study used 10 single models in the biomod2 algorithm, including the generalized additive model (GAM), the generalized boosted regression model (GBM), the generalized linear model (GLM), classification tree analysis (CTA), surface range envelope (SRE), artificial neural network (ANN), multivariate adaptive regression splines (MARS), flexible discriminant analysis (FDA), the maximum entropy (MaxEnt) model, and random forest (RF) (Thuiller et al., 2022). For model setup and evaluation, 10,000 random pseudo-absence records were generated for the 10 modeling algorithms on a global scale (excluding Antarctica) (Inman et al., 2021). Of the species distribution records, 75% were randomly selected as the training set, and the remaining 25% were used as the testing set. The model setup was run five times and ultimately produced 50 predictions to ensure the stability of model predictions. The area under the receiver operating characteristic (ROC) curve (AUC) and the true skill statistic (TSS) values were used to assess the simulation accuracy of the model (Allouche et al., 2006; Landgrebe and Duin, 2008). The values of AUC and TSS range from 0 to 1 and -1 to 1, respectively; the larger the value, the better the model prediction. We selected single models with AUC values greater than 0.9 and TSS values greater than 0.8 to construct the ensemble model (**Supplementary Table S2**).

### 2.3 Classification of suitable areas and their overlapping areas with hosts

The prediction result of the ensemble model was an ASCII file representing the species’ existence probability; the larger the value, the higher the probability of the species’ existence. Suitable areas for the species were classified into four categories using the reclassification tool in ArcGIS. For *E. amylovora*, the four categories were: unsuitable area (0–0.14), lowly suitable area [0.14–0.4), moderately suitable area [0.4–0.6), and highly suitable area [0.6–1).

The main hosts affected by *E. amylovora* are pears and apples. Therefore, global production areas of pears and apples were obtained from the EARTHSTAT website (http://www.earthstat.org/harvested-area-yield-175-crops/). The host production area data represent the harvested area data for pears and apples in the year 2000. These data are the average values of the harvested area within the grid cell for pears and apples during the period from 1997 to 2003. The suitable areas for *E. amylovora* were overlapped with the host production areas in ArcGIS to obtain the overlapping areas.

### 2.4 Reconstruction of dispersal history and routes

The dispersal histories of *E. amylovora* from 2015 to 2022 were reconstructed and analyzed using county-scale species occurrence areas as the basic spatial unit in China. The occurrence area data were primarily obtained from published literature and the List of Agricultural Plant Quarantine Pests Distribution Administrative Areas in China. To better understand the dispersal patterns of *E. amylovora* in China, the locality center of the occurrence area was calculated at the county scale. These locality centers were used to calculate the dispersal distance and to reconstruct the dispersal routes in China using the Euclidean distance. Specifically, the minimum cost arborescence approach in the ecospat package was used to reconstruct the dispersal routes of *E. amylovora* (Hordijk and Broennimann, 2012), calculate the minimum, observed, and random dispersal route lengths for 2015–2022, and perform 1,000 iterations (Di Cola et al., 2017). Finally, a histogram was created to compare the calculated heterogeneity between the three total dispersal route lengths.

### 2.5 Identification of the dispersal risk areas

To identify the dispersal risk areas of *E. amylovora* in China based on the occurrence areas from 2015 to 2022 and the potential suitable areas under near-current climatic conditions, we modeled the dispersal processes of *E. amylovora* from its initial distribution area (the overlapping areas of occurrence and potential suitable areas) to its potential distribution under near-current climatic conditions. The probabilities of *E. amylovora* dispersal to different areas of distance were calculated by constructing a dispersal kernel. The probabilities of *E. amylovora* dispersal to different distances were negatively exponential with the distance. The dispersal probability was calculated using the following formula 1:

(1) $$P(d)=e^{-\text{d}/l}$$

where *P*(*d*) denotes the probability of *E. amylovora* dispersal to a spatial distance (*d*) and *l* indicates the dispersal distance of *E. amylovora*. The short-distance dispersal of *E. amylovora* is 16 km/year, while its long-distance dispersal is 100 km/year (Hu and Xu, 1999). The potential suitable areas for *E. amylovora* in China are 2.5 arcmin (≈5 km) in resolution. Thus, dispersal events with a distance ≤4 raster units were set as the short-distance dispersal events, while those with >4 raster units were set as the long-distance dispersal events. We set the frequency of the long-distance dispersal of *E. amylovora* to 0.8 and simulated the dispersal process of the species in China using the MigClim package (Engler and Guisan, 2009).

### 2.6 Economic loss assessment model

The economic impacts of *E. amylovora* on the pear and apple industries in China were assessed, including the no-control scenario and the control scenario. The no-control scenario mainly includes yield losses and quality losses caused by *E. amylovora* to the pear and apple industries. The control scenario includes losses after control and control costs. Potential economic losses under the no-control scenario (F_3_) include economic losses due to production decline (F_1_) and quality decline (F_2_). Economic losses under the control scenario (F_6_) include control costs (F_4_) and economic losses after control measures (F_5_). The economic loss savings after control would be the difference between the potential economic loss under the no-control scenario (F_3_) and the control scenario (F_6_). The economic losses that can be saved after control measures are F_7_. The calculation formulas 2-4 are as follows:

(2) $${\text{F}}_{3}={\text{F}}_{1}+{\text{F}}_{2}$$

(3) $${\text{F}}_{6}={\text{F}}_{4}+{\text{F}}_{5}$$

(4) $${\text{F}}_{7}={\text{F}}_{3}-{\text{F}}_{6}$$

The potential economic loss formulas and the parameters for each component are presented in “Materials and methods” of the *Electronic supplementary material*. The specific nodes, the input variables, and the parameters in the model for assessment of the potential economic losses due to *E. amylovora* to the host industry are shown in **Supplementary Tables S3** and **S4**. The collated model parameters were entered into @RISK software (version 8.5.2), and the number of model iterations was set to 200,000. The sensitivity analysis feature of the software was also used to identify the key input variables affecting the output results (Kang et al., 2019).

## 3 Results

### 3.1 Global potential geographic distribution of *E. amylovora* under near-current and future climatic conditions

**Figure 2:**
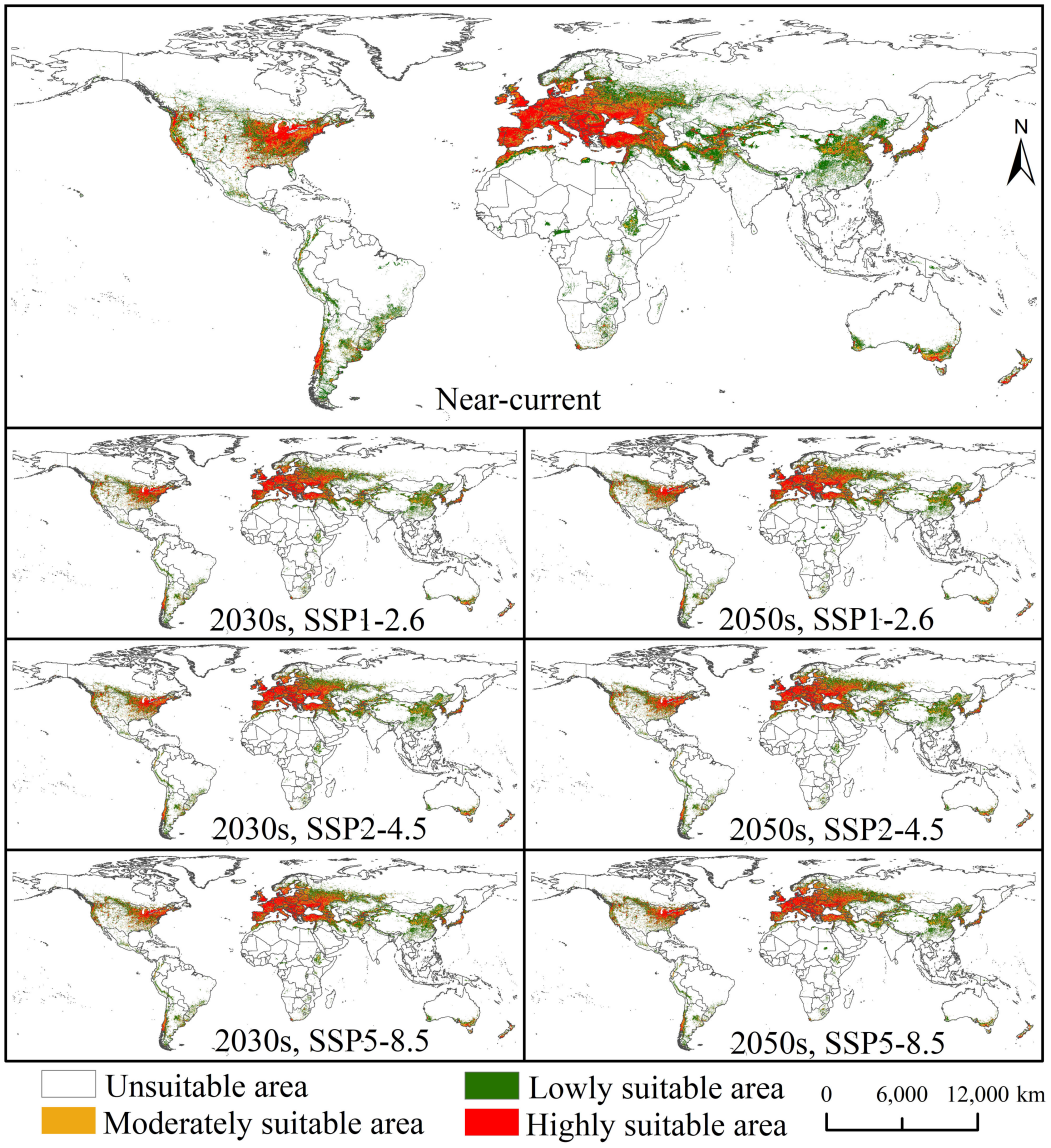
Potential suitable areas for *Erwinia amylovora* globally under near-current and future climatic conditions.

The results showed that the total suitable area globally for *E. amylovora* is 2,614.26 × 10^4^ km^2^ under near-current climatic conditions (**Supplementary Table S5**), mainly located in North America, southern South America, Europe, northern and southern Africa, western and eastern Asia, and southern Oceania (**Figure 2**). The highly suitable area of *E. amylovora* is 800.69 × 10^4^ km^2^, which is mainly located in central North America (United States), southern South America (southern Chile), Europe (Spain, Portugal, France, United Kingdom, Germany, Netherlands, Belgium, Switzerland, Italy, Austria, Slovenia, Poland, Czech Republic, Slovakia, Romania, southwestern Russia, Ukraine, Greece, Serbia, and Bulgaria), western and eastern Asia (Turkey, Iran, Afghanistan, China, South Korea, and Japan), northern Africa (northern Morocco and northern Algeria), and Oceania (southern Australia and New Zealand). The moderately suitable area is 554.43 × 10^4^ km^2^, mainly located in central North America (United States), southern South America (Argentina and Chile), central Europe (Poland, Ukraine, and western Russia), western and eastern Asia (Turkey, Iran, Japan, and China), and southern Oceania (Australia). The lowly suitable area is 1259.14 × 10^4^ km^2^, mainly located in central North America (Canada and the United States), central South America (southern Brazil, Argentina, and Peru), eastern Europe (western Russia), central and southern Africa (Ethiopia and South Africa), western and central Asia (Iran, Afghanistan, and China), and southern Australia. The areas of the total suitable area are 2,653.65, 2,649.09, and 2,700.93 × 10^4^ km^2^ under the SSP1-2.6, SSP2-4.5, and SSP5-8.5 scenarios, respectively, for the 2030s and are 2,693.22, 2,705.39, and 2,714.93 × 10^4^ km^2^, respectively, for the 2050s (**Supplementary Table S5**). However, *E. amylovora* showed no significant changes in the distribution areas under future climate conditions compared with near-current climate conditions (**Figure 2**). The moderately and lowly suitable areas showed an increasing trend under future climatic conditions, whereas the highly suitable areas showed a decreasing trend. Under future climatic conditions, the increase areas of *E. amylovora* will mainly be located in western Russia, northwestern and northeastern China, northern Kazakhstan, southern Finland, southern Canada, and northern United States (**Supplementary Figure S2**).

### 3.2 Overlapping areas between the potential suitable areas for *E. amylovora* and host production areas under near-current and future climate conditions

**Figure 3:**
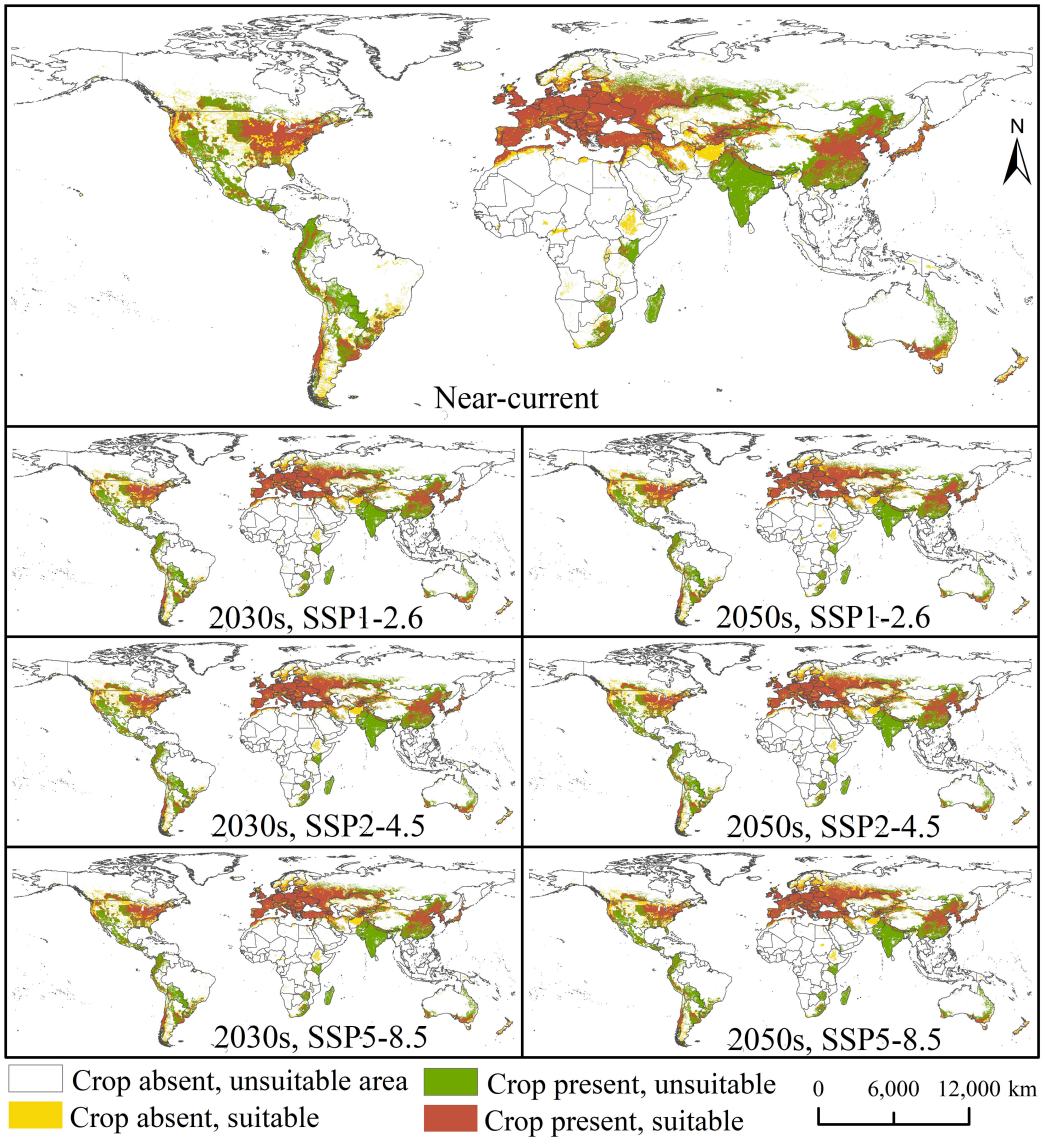
Potential suitable areas for *Erwinia amylovora* overlapping with the host production areas under near-current and future (2030s and 2050s, respectively) climatic conditions (SSP1-2.6, SSP2-4.5, and SSP5-8.5).

**Figure 3** shows the overlap between the potential suitable areas for *E. amylovora* and the host production areas worldwide under the near-current and future (2030s and 2050s) climatic conditions. The area of *E. amylovora* overlapping with the host (pear and apple) production area is 1,897.62 × 10^4^ km^2^ under near-current (**Supplementary Table S5**) climatic conditions, accounting for 53.11% of the main host production area. This region is mainly located in central North America (the United States), southern South America (eastern Argentina, Chile, and Uruguay), Europe (almost the entire continent), northern Africa (northern Morocco, northern Algeria, and South Africa), western and eastern Asia (Turkey, Georgia, Azerbaijan, Iran, Kazakhstan, China, the Korean Peninsula, and Japan), and southern Oceania (southern Australia) (**Figure 3**). The overlapping areas are 1,961.42, 1,955.49, and 1,991.83 × 10^4^ km^2^ under the SSP1-2.6, SSP2-4.5, and SSP5-8.5 scenarios, respectively, for the 2030s and are 1,982.18, 1,991.56, and 1,984.66 × 10^4^ km^2^ for the 2050s. The potential geographic distribution of *E. amylovora* and its overlapping areas with the host production areas will change slightly compared with those of the near-current climatic conditions and will show increasing trends.

### 3.3 Historical process dispersal, dispersal routes, and dispersal risk areas of *E. amylovora*

**Figure 4:**
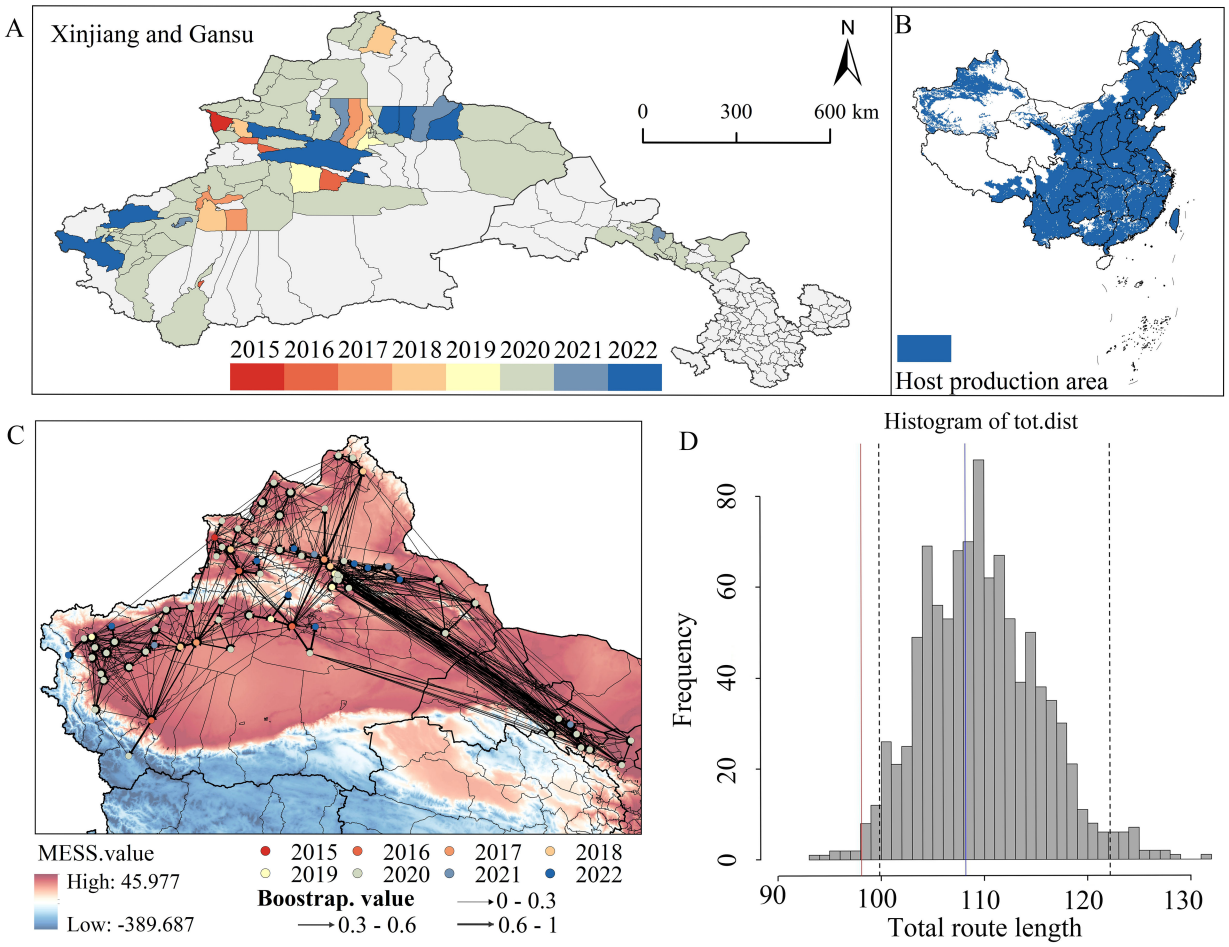
**(A)** Historical dispersal process of *Erwinia amylovora* at the county scale. Xinjiang and Gansu are the occurrence areas of *E*. *amylovora*. **(B)** Distribution of the main hosts (pear and apple) production areas in China. **(C)** Dispersal route of *E*. *amylovora* in China from 2015 to 2022. **(D)** Histogram of the total paths for 1,000 randomly assigned values. *Dashed lines* indicate the 0.05 and 0.95 quantiles. Blue indicates the observed values and red the minimum values for *E*. *amylovora.*.

Within 2015–2022, the occurrence area of *E. amylovora* was mainly distributed in Xinjiang and Gansu (**Figure 4A**). The species was first discovered in China in 2015 and was subsequently dispersed within Xinjiang and introduced into the cities of Zhangye and Wuwei, Gansu Province, after 2020. Simultaneously, the number of occurrence counties increased, particularly in 2020. The current distribution area of *E. amylovora* overlaps with the host production areas mainly in northwestern China (**Figure 4B**). From 2015 to 2022, the distribution range of *E. amylovora* in China covered areas with climates similar to the habitats of the United States population, as well as areas with significantly different climates (**Figure 4C**). The total route lengths obtained using the observation method were within the 5%–95% quantiles of the total route lengths obtained using the random reassignment method, whereas the total route lengths obtained using the minimum method were outside the interval (**Figure 4D**). This suggests that the dispersal pattern of *E. amylovora* in China is mainly a “leap-frog” long-distance dispersal.

**Figure 5:**
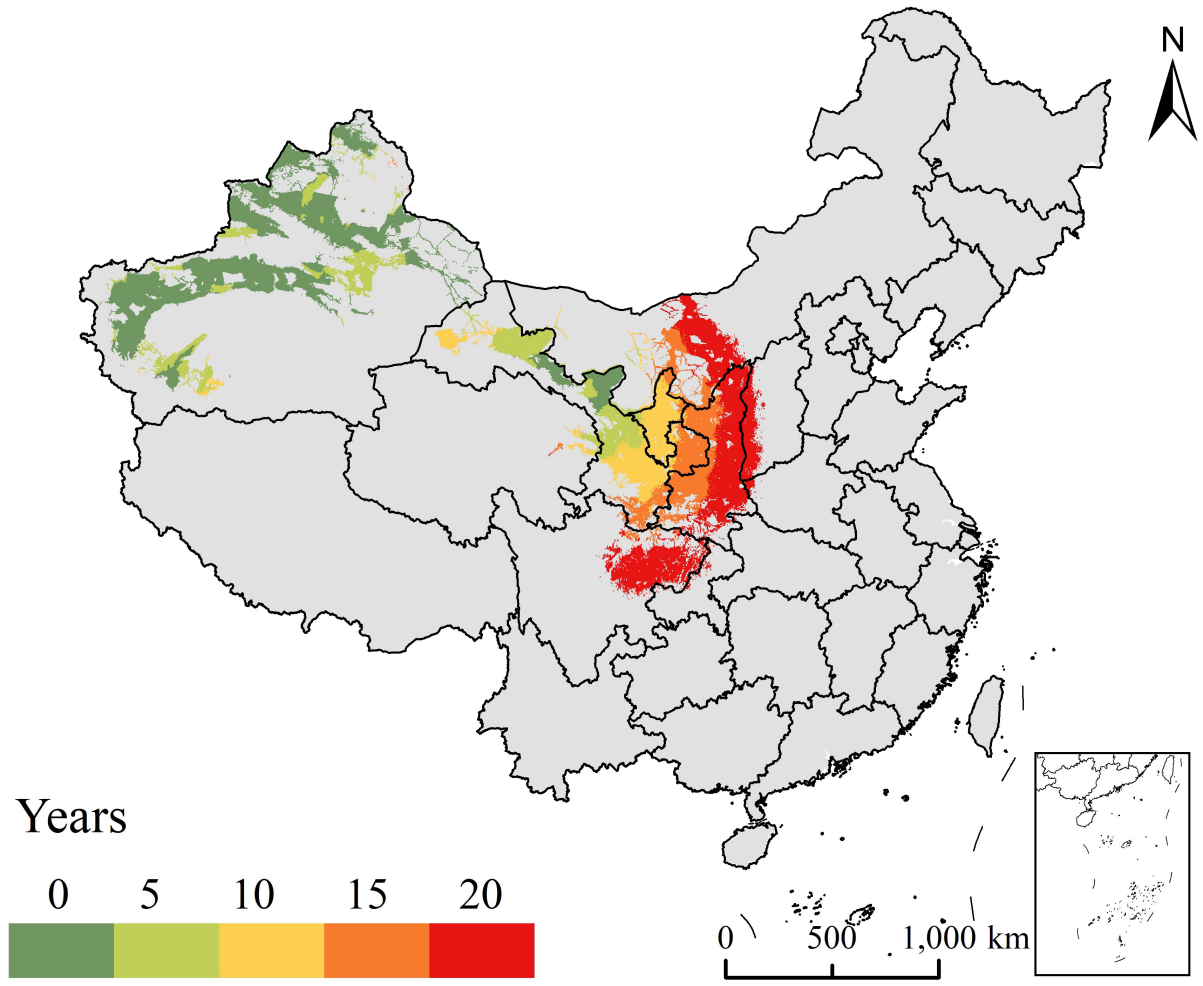
Trends in dispersal risk areas of *Erwinia amylovora* from the initial distribution areas to the potential distribution areas during different periods in China.

The distributions of *E. amylovora* in China showed expansion trends under the near-current climatic conditions. The dispersal areas of *E. amylovora* from its initial distribution to potential suitable areas were mainly in Xinjiang and Gansu in the 1-5 years, Ningxia and southeastern Gansu in 6–10 years, Shaanxi and parts of Gansu in 11–15 years, and Shaanxi, Sichuan, and Inner Mongolia in 16–20 years (**Figure 5**).

### 3.4 Potential economic losses under different control scenarios

**Figure 6:**
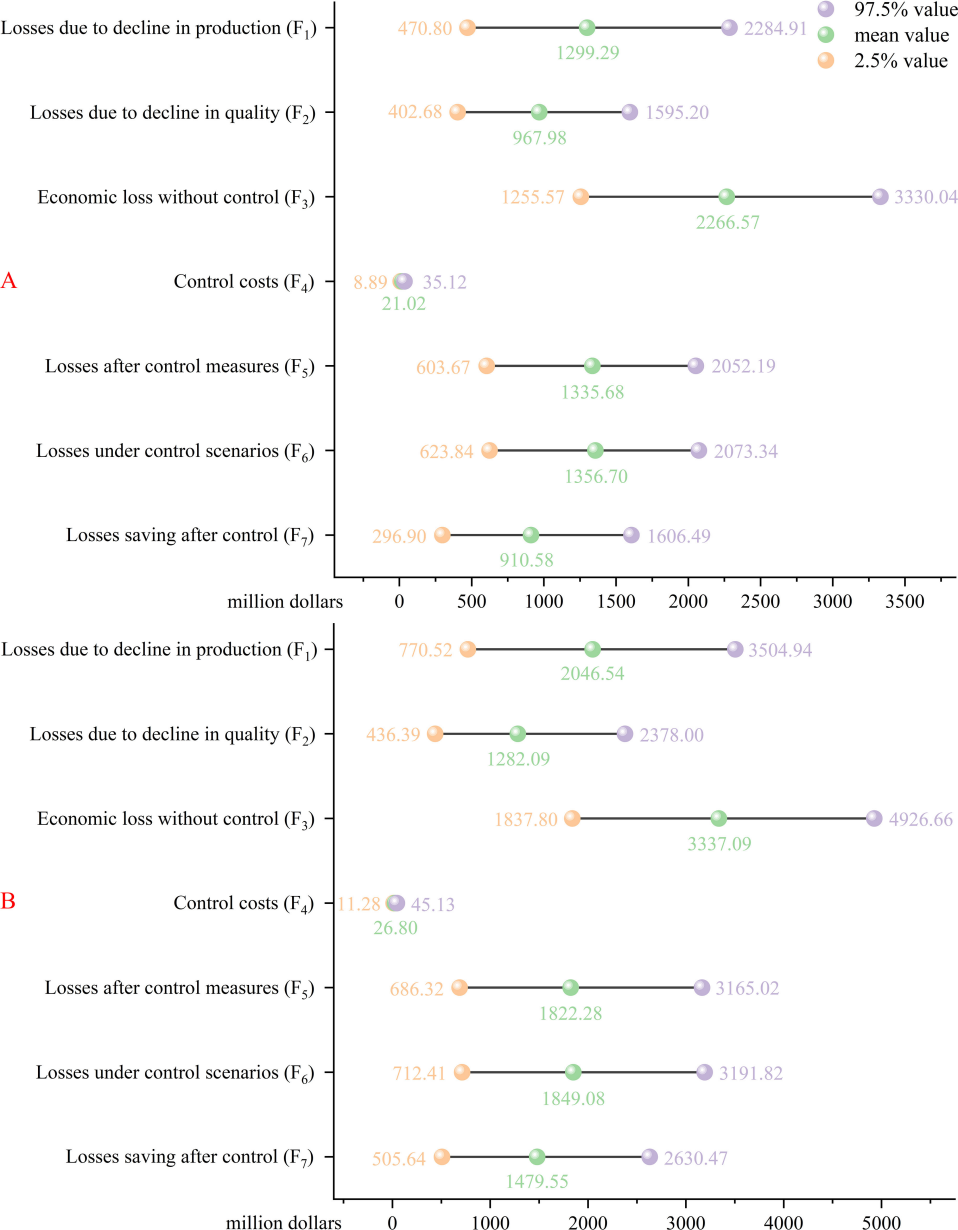
Potential economic losses to the pear **(A)** and apple **(B)** industries in China due to *Erwinia amylovora* under near-current climatic conditions (in million dollars). The economic losses under the no-control scenario are as follows: *F_1_* represents the losses due to a decline in production, *F_2_* represents the losses due to a decline in quality, and *F_3_* represents the economic losses without control. The economic losses under the control scenario are as follows: *F_4_* represents the control costs, *F_5_* represents the losses after control measures, and *F_6_* represents the losses under the control scenario. *F_7_* represents the losses saved after control.

Under the near-current climatic conditions, the potential economic losses caused by *E. amylovora* to the pear industry in China under the no-control scenario would be 1,255.57–3,330.04 million dollars (95% confidence level), with a mean value of 2,266.57 million dollars (**Figure 6A**). The mean economic losses due to a decline in production and quality would be 1,299.29 and 967.98 million dollars, respectively. The potential economic losses under the control scenario (chemical control measures) would be 623.84–2,073.34 million dollars (95% confidence level), with a mean value of 1,356.70 million dollars. The mean control costs and economic losses after control measures would be 21.02 and 1,335.68 million dollars, respectively. The potential economic losses to be saved under control conditions would be 296.90–1,606.49 million dollars (95% confidence level), with a mean value of 910.58 million dollars.

The potential economic losses caused by *E. amylovora* to the apple industry in China under the no-control scenario would be 1837.80–4,926.66 million dollars (95% confidence level), with a mean value of 3,337.09 million dollars (**Figure 6B**). The mean economic losses due to a decline in production and quality would be 2,046.54 and 1,282.09 million dollars, respectively. The potential economic losses under the control scenario would be 712.41–3,191.82 million dollars (95% confidence level), with a mean value of 1,849.08 million dollars. The mean control costs and the economic losses after control measures would be 26.80 and 1,822.28 million dollars, respectively. The potential economic losses to be saved under the control conditions would be 505.64–2,630.47 million dollars (95% confidence level), with a mean value of 1,479.55 million dollars. The input values affecting the potential economic losses caused by *E. amylovora* are mainly the host price (*P*_a_ and *P*_b_), the host production loss rate (*R*), and the host damage rate by species (*I*) (**Supplementary Figure S3**).

## 4 Discussion

The occurrence of *E. amylovora* usually causes large-scale death of its hosts, resulting in significant yield and economic losses to the pear and apple industries (Ham et al., 2022; Huang et al., 2022). Understanding the establishment risk of *E. amylovora* in host production areas globally, as well as the localized dispersal risk areas and economic losses, can help in the development of targeted control measures and accurate monitoring. Therefore, this study identified potential suitable areas for the species and areas overlapping with the host production areas at the global scale, subsequently identified the dispersal patterns with dispersal risk areas of the target species in China, and finally evaluated the economic impacts on the hosts.

Our results indicate that *E. amylovora* is mainly distributed in North America, southern South America, Europe, northern Africa, western and eastern Asia, and southern Oceania under the near-current climatic conditions. *Erwinia amylovora* is mainly located in temperate continental, temperate monsoon, and temperate oceanic climates. This is consistent with the suitable temperature, precipitation, and seasonal climatic conditions required for the distribution of the species. The flower stigma is the only stage at which *E. amylovora* reproduces on the plant surface. Temperatures between 18°C and 30°C, with rainfall during host flowering, favor flower infection with *E. amylovora* (CABI, 2024). The climatic conditions during the spring and summer play a key role in the occurrence and development of *E. amylovora* (Wu et al., 2023). The research results also indicate that the distribution areas of *E. amylovora* will expand under future climatic conditions. Climate change can alter the distribution and population abundance of plant pathogens owing to changes in temperature and humidity (Ajene et al., 2020; Kueneman et al., 2019), which directly affect the growth, reproduction, and infection of pathogens. Under field conditions, the epidemiology of *E. amylovora* requires temperatures above 18°C, and the expression of pathogenicity and other functional genes is enhanced with increasing temperatures, with the optimal growth temperature being at 28°C (Wei et al., 1992; Goyer and Ullrich, 2006; Santander and Biosca, 2017). Moreover, some studies have shown that the population of *E. amylovora* increased more than 10-fold in 24h at temperatures ranging from 15°C to 34°C (Smith and Pusey, 2011). Our results indicate that the potential distribution area of *E. amylovora* and the host production areas exhibit a high degree of overlap. The target species are basically distributed among the major pear- and apple-growing countries worldwide (FAO, 2024). For example, the damage and control costs of *E. amylovora* in the United States have been estimated to exceed US $100 million annually (Norelli et al., 2003). Pears and apples also play important roles in local disease outbreaks (Sun et al., 2023). After the introduction of *E. amylovora* into Yili City of Xinjiang in northwestern China in 2015, it has spread to the majority of the pear and apple production areas in Xinjiang and parts of Gansu Province. The northwest region is an important apple and pear growing region in China, with Xinjiang and Gansu ranking in the top 10 for production in the country. Therefore, under climate change conditions, countries around the globe should strengthen quarantine and control in the potential distribution areas and host damage areas of *E. amylovora* in order to reduce the risk and stress of establishment globally.

The dispersal history of *E. amylovora* shows that its occurrence has increased in China from 2015 to 2022. The outbreak and epidemic of fire blight may be related to multiple factors, including susceptible hosts, low disease resistance, suitable climate, and various carriers (Hu and Xu, 1999; Chen et al., 2022; Li et al., 2022). The majority of pears and apples grown in China, including *Pyrus sinkiangensis* Yü (Korla fragrant pear), *Pyrus bretschneideri* (Yali), and *Malus domestica* Borkh. cv. Fuji (Red Fuji), are susceptible varieties (Sun et al., 2023; Fang et al., 2022; Wu et al., 2018). In addition, majority of the pear and apple varieties in Xinjiang have low resistance to *E. amylovora* (Chen et al., 2022; Li et al., 2022). Fire blight is prone to outbreaks during suitable temperatures and rainfall conditions. For example, during the pear flowering season from 2017 to 2021, the years with higher rainfall corresponded to more severe disease outbreaks in Korla City, while the years with lower rainfall had infection rates below average (Sun et al., 2023). The long-distance dispersal of *E. amylovora* mainly relies on host plants, plant tissues, and packaging materials to spread across provinces, countries, and even continents (Hu and Xu, 1999; Sun et al., 2023). Insects (bees), birds, wind, and rain are important factors that affect short-distance dispersal. For example, the proportion of orchards infected with *E. amylovora* through bee pollination is approximately six times higher than when bee pollination is prohibited (Ban et al., 2023). Research results indicate that *E. amylovora* is dispersed mainly through “leap-frog” long-distance dispersal in China from 2015 to 2022. This means that China faces a greater dispersal risk of *E. amylovora* and should be concerned about the direction of the next dispersal risk, including central Inner Mongolia, Shaanxi, western Shanxi, Xinjiang, Gansu, Ningxia, and eastern Sichuan. Therefore, these regions should impose stricter quarantine requirements on susceptible hosts from neighboring risk areas, in particular Shaanxi, the province with the highest apple production in China and with the susceptible apple variety ‘Red Fuji’ (Fang et al., 2022; Sun et al., 2023).

China ranks first globally in the production of apples and pears, which are also the second and third most consumed fruits in the country, respectively (Sun et al., 2023). *Erwinia amylovora* is among the major economic threats to the pear and apple industries in recent years. Under the no-control scenario, *E. amylovora* could cause economic losses of 2,266.57 and 3,337.09 million dollars to the pear and apple industries, respectively. Moreover, *E. amylovora* could cause economic losses of 1,356.70 and 1,849.08 million dollars to the pear and apple industries, respectively, under the control scenario. However, economic losses can be significantly reduced through control measure. Cook et al. (2011) used @RISK software to assess the possible economic losses of due to *E. amylovora* to Australian imports of New Zealand apples from both trade revenues and the cost of invasive species outbreaks. Our study only assessed the potential economic losses caused by *E. amylovora* to the pear and apple industries in China and did not consider trade incomes, mainly because the study species is already distributed in China. Previous studies have used @RISK software to assess the potential economic impact caused by this invasive species, mainly focusing on the economic losses that may be caused by its geographical distribution (Fang et al., 2015; Kang et al., 2019; Xi et al., 2022). The variables with the greatest impact on economic losses included the prices of pears and apples, the host infection rate, and the yield loss rate after host infection. The infection rates of *E. amylovora* on pears and apples and the resulting rates of yield loss were mainly derived from field surveys; therefore, the economic loss results are more accurate and reliable. Although price factors exert the most significant influence on potential economic losses, their artificial adjustment remains challenging. Therefore, the economic losses should be reduced by decreasing the damage rate and the loss rates of production of *E. amylovora* to the hosts. Here, countries worldwide should strengthen the quarantine of *E. amylovora*, block the dispersal pathway, and monitor the dispersal risk areas in China to minimize the infection and harm to the hosts.

This study reveals the establishment risk of *E. amylovora* globally and its hazard risk in the host production areas while analyzing its dispersal patterns, dispersal risk areas, and economic impacts in China. The results of this study can provide guidance for the prevention, control, and early warning for *E. amylovora* worldwide while curbing its dispersal risk in China and reducing its negative impacts on the pear and apple industries. However, this study has some limitations. Firstly, the distribution records of *E. amylovora* were mainly obtained from websites, the literature, and some field surveys. More field surveys could be conducted in the future to further improve the prediction accuracy. Secondly, the pear and apple production area data were from the 2000s. Subsequent studies should attempt to obtain more recent data in order to enhance temporal validity. Finally, more factors should be incorporated into species dispersal studies, such as species abundance, transportation, and other indicators, to improve the reliability of the predictions.

## 5 Conclusions

This study comprehensively assessed the establishment risk of *E. amylovora* in host production areas globally, as well as the dispersal trends and the potential economic losses to the pear and apple industries in China. The results showed that *E. amylovora* is mainly distributed in North America, southern South America, Europe, northern and southern Africa, western and eastern Asia, and southern Oceania under the near-current climatic conditions. The global pear and apple production area face a significant threat from *Erwinia amylovora*, which could worsen under future climatic conditions. *Erwinia amylovora* shows mainly a “leap-frog” long-distance dispersal in China, which will face a greater dispersal risk and threat. *Erwinia amylovora* dispersal risk areas primarily include central Inner Mongolia, Shaanxi, western Shanxi, Xinjiang, Gansu, Ningxia, and eastern Sichuan. Under the no-control scenario, *E. amylovora* could cause mean losses of 2,266.57 and 3,337.09 million dollars to the pear and apple industries in China, respectively. Under control measures, its mean losses were 1,356.70 million dollars and 1,849.08 million dollars, respectively. Global countries should strengthen the prevention, control, and monitoring of *E. amylovora*, particularly those areas where increases are expected under future climate conditions. This study provides a theoretical basis for the development of priority management strategies for *E. amylovora* to improve precision interception and monitoring measures, thereby reducing potential impacts on the pear and apple industries.

## Data Availability

The raw data supporting the conclusions of this article will be made available by the authors, without undue reservation.
