# Supplementary material for: Comprehensive assessment of Erwinia amylovora: from establishment risk in global host production areas to dispersal dynamics and associated economic losses in China
Source: Front Plant Sci. 2026 Feb 4;16:1641129. doi: 10.3389/fpls.2025.1641129 (PMC12913581; doi:10.3389/fpls.2025.1641129)
Supplement: Supplementary file 1 [file DataSheet1.docx]

Supplementary Material

Comprehensive assessment of *Erwinia amylovora*: from establishment risk in global host production areas to dispersal dynamics and associated economic losses in China

Ming Li ^1 †^, Xiaoqing Xian ^1 †^, Zhenan Jin ^1^, Yuhan Qi ^1^, Jianyang Guo ^1^, Nianwan Yang ^1,2^, Guifen Zhang ^1^, Jin Xu ^1 *^, Wanxue Liu ^1 *^

^1^ State Key Laboratory for Biology of Plant Diseases and Insect Pests, Key Laboratory for Prevention and Control of Invasive Alien Species of Ministry of Agriculture and Rural Affairs, Institute of Plant Protection, Chinese Academy of Agricultural Sciences, Beijing 100193, China;

^2^ Western Agricultural Research Center, Chinese Academy of Agricultural Sciences, Changji 831100, China

*** Correspondence:**Wanxue Liu; Jin Xu,
liuwanxue@caas.cn; jinxu@ippcaas.cn

^†^ These authors have contributed equally to this work

**Supplementary materials and methods**

The F_1_ and F_2_ are the potential economic losses due to production decline and quality decline, respectively, under the no-control scenario. The F_4_ and F_5_ are the control costs and potential economic losses caused after control measures, respectively, under the control scenario. The formulas are as follows:

|  | F_1_ = Q_1_×I×R×Pa/(1-IR) | (1) |
| --- | --- | --- |
|  | F_2_ = Q_1_×I×(1-R) ×(Pa-Pb)/(1-IR) | (2) |
|  | F_4_ = S×I×C | (3) |
|  | F_5_ = Q_1_×I×E×Pa/(1-IE) + Q_1_×I×(1-E) ×(Pa-Pb)/ (1-IE) | (4) |

In the formula, Q_1_ and S represent the annual production and harvest area of the host in the suitable area of species, respectively. These values are obtained by first calculating the ratio of overlapping area between the suitable area of species and the production area of hosts to the total area of hosts, and then multiplying it with the annual production and annual harvest area of hosts. The pears and apples annual production and harvest area were obtained from the Food and Agriculture Organization of the United Nations (FAO, 2024) in China from 2018 to 2022. I denote the damage rate of the *E. amylovora* to the host, R is the yield loss rate of the host after damage by the species, C is the unit control cost, and the above data are from the data in the national field survey. Pa and Pb are the market normal price of the host and the market price after host damage, respectively, from the National Key Agricultural Products Market Information Platform (https://ncpscxx.moa.gov.cn). The collected host price data was divided into two parts from largest to smallest; the larger half of the data was selected as the market normal price of the host, and the smaller half as the market price of the host after the damage.

E is the economic injury level, which is the yield loss rate of the host after control, and the formulas are as follows:

|  | E = C/(A×Pa×M) ×D×100% | (5) |
| --- | --- | --- |
|  | A = Q_1_/S | (6) |

In the formula, A is the unit yield of the host in the suitable area. D is the control measure (chemical control) effect, which was obtained from literature (Wang et al., 2022; Bai et al., 2023). D is the efficiency correction coefficient, which is generally considered to be two times the benefit of the expenditure (Pan et al., 2014), and in this study D is 2.

Bai, J., Zhu, H. H., Zhang, G. Q. (2023). Preliminary Screening of Control Agents and Exploration of Optimum Fire Blight Control Period in Korla. *Xinjiang Agricultural Sciences*. 60, 675-682.

FAO, (2024). Food and Agriculture Organization of the United Nations.

Pan, F., Xiao, T. B., Qin, S., Chen, H. Y., Lin, Z. F., Xie, S. H. (2014). Study on yield of bitter gourd damaged by *Bactrocera cucurbitae* and its economic thresholds. *China Plant Protection*. 34, 12-15.

Wang, J. H., Niu, Y. L., Qin, J. G., Han, L. L., Chen, W. M. (2022). Field control effect and evaluation of four fungicides on fire blight of pear. *Agrochemicals*. 61, 523-525.

**Supplementary Tables**

Table S1. Environmental variables for constructing species distribution models of *Erwinia amylovora*.

| Variables | Description | Unit | Whether to construct model |
| --- | --- | --- | --- |
| Bio1 | Annual mean temperature | ◦C | Yes |
| Bio2 | Mean diurnal temperature area | ◦C | Yes |
| Bio3 | Isothermality (bio2/bio7) | - | Non |
| Bio4 | Temperature seasonality | - | Non |
| Bio5 | Max temperature of warmest month | ◦C | Non |
| Bio6 | Min temperature of coldest month | ◦C | Non |
| Bio7 | Temperature annual area (bio5-bio6) | ◦C | Yes |
| Bio8 | Mean temperature of wettest quarter | ◦C | Yes |
| Bio9 | Mean temperature of driest quarter | ◦C | Non |
| Bio10 | Mean temperature of warmest quarter | ◦C | Non |
| Bio11 | Mean temperature of coldest quarter | ◦C | Non |
| Bio12 | Annual precipitation | mm | Yes |
| Bio13 | Precipitation of wettest month | mm | Non |
| Bio14 | Precipitation of driest month | mm | Yes |
| Bio15 | Precipitation seasonality | - | Yes |
| Bio16 | Precipitation of wettest quarter | mm | Non |
| Bio17 | Precipitation of driest quarter | mm | Non |
| Bio18 | Precipitation of warmest quarter | mm | Yes |
| Bio19 | Precipitation of coldest quarter | mm | Yes |
| Altitude | Altitude | m | Yes |
| Hii | Human influence index | - | Yes |

Table S2. Mean values under receiver operating characteristic (ROC) curves (AUC) and true skill statistics (TSS) for *Erwinia amylovora*.

| Models | AUC | TSS | |
| --- | --- | --- | --- |
| EM | 0.962 | | 0.843 |
| ANN | 0.888 | | 0.730 |
| CTA | 0.920 | | 0.804 |
| GAM | 0.954 | | 0.810 |
| SRE | 0.770 | | 0.541 |
| FDA | 0.953 | | 0.796 |
| GBM | 0.967 | | 0.835 |
| GLM | 0.961 | | 0.839 |
| MARS | 0.961 | | 0.837 |
| MaxEnt | 0.957 | | 0.817 |
| RF | 0.973 | | 0.862 |

Table S3. Assessment of potential economic loss models and parameters of *Erwinia amylovora* to the pear industry.

| Scenarios | Evaluation items | Input variable | Model parameters |
| --- | --- | --- | --- |
| no-control scenarios | Losses due to decline in production-F_1_ = Q_1_×I×R×Pa/(1-IR) | pear production in suitable areas-Q_1_, ×10^8^ kg | Pert (94.15,105.07,112.58) |
|  |  | damage rates of *E. amylovora* to pears-I | Pert (28.2%,29.9%,31.6%) |
|  |  | loss rates of production of pears after damage-R | Pert (5%,32.5%,60%) |
|  |  | the market price of pears -Pa, dollars/kg | Pert (0.82,1.16,1.41) |
|  | Losses due to decline in quality-F_2_ = Q_1_×I×(1-R)×(Pa-Pb)/(1-IR) | price of pears after the quality decline-Pb, dollars/kg | Pert (0.55,0.75,0.82) |
|  | Economic loss without control-F_3_ | | F_3_ = F_1_+ F_2_ |
| control scenarios | control costs-F_4_ = S×I×C | harvested area of pears in suitable areas-S, ×10^4^ hm^2^ | Pert (55.15,56.62,58.40) |
|  |  | unit cost of control-C, dollars/ hm^2^ | Pert (31.73,114.37,253.88) |
|  | Losses after control measures-F_5_ = Q_1_×I×E×Pa/(1-IE)+ Q_1_×I×(1-E)×(Pa-Pb)/ (1-IE) | unit yield of pears-A, kg/ hm^2^ | A = Q_1_/S |
|  |  | control effects-M | Pert (78.6%,87.4%,96.2%) |
|  |  | efficiency correction coefficient-D | 2 |
|  |  | economic injury level-E | E=C/(A×Pa×M)×D×100% |
|  | Losses under control scenarios-F_6_ | | F_6_ = F_4_+ F_5_ |
|  | Losses saving after control-F_7_ | | F_7_= F_3_-F_6_ |

Table S4. Assessment of potential economic loss models and parameters of *Erwinia amylovora* to the apple industry.

| Scenarios | Evaluation items | | Input variable | Model parameters |
| --- | --- | --- | --- | --- |
| no-control scenarios | Losses due to decline in production-F_1_ = Q_1_×I×R×Pa/(1-IR) | | apple production in suitable areas-Q_1_, ×10^8^ kg | Pert (199.73,223.26,242.17) |
|  |  |  | damage rates of *E. amylovora* to apples-I | Pert (18.20%,20.7%,23.20%) |
|  |  |  | loss rates of production of apples after damage-R | Pert (5%,32.5%,60%) |
|  |  |  | the market price of apples -Pa, dollars/kg | Pert (1.11,1.23,1.58) |
|  | Losses due to decline in quality-F_2_ = Q_1_×I×(1-R)×(Pa-Pb)/(1-IR) | | price of apples after the quality decline-Pb, dollars/kg | Pert (0.14,0.92,1.11) |
|  | Economic loss without control-F_3_ | | | F_3_ = F_1_+ F_2_ |
| control scenarios | control costs-F_4_ = S×I×C | | harvested area of apples in suitable areas-S, ×10^4^ hm^2^ | Pert (98.69,104.82,108.38) |
|  |  |  | unit cost of control-C, dollars/ hm^2^ | Pert (31.73,114.37,253.88) |
|  | Losses after control measures-F_5_ = Q_1_×I×E×Pa/(1-IE)+ Q_1_×I×(1-E)×(Pa-Pb)/ (1-IE) | | unit yield of apples-A, kg/ hm^2^ | A = Q_1_/S |
|  |  |  | control effects-M | Pert (78.6%,87.4%,96.2%) |
|  |  |  | efficiency correction coefficient-D | 2 |
|  |  |  | economic injury level-E | E=C/(A×Pa×M)×D×100% |
|  | Losses under control scenarios-F_6_ | | | F_6_ = F_4_+ F_5_ |
|  | | Losses saving after control -F_7_ | | F_7_= F_3_-F_6_ |

Table S5. Area of each suitable area and overlapping areas with host production areas for *Erwinia amylovora* (hosts are pear and apple) under near current and future climatic conditions (10^4^ km^2^).

| periods | lowly | moderately | highly | total | overlapping area |
| --- | --- | --- | --- | --- | --- |
| current | 1259.14 | 554.43 | 800.69 | 2614.26 | 1897.62 |
| 2030s, SSP1-2.6 | 1265.72 | 586.80 | 801.13 | 2653.65 | 1961.42 |
| 2030s, SSP2-4.5 | 1264.12 | 588.09 | 796.88 | 2649.09 | 1955.49 |
| 2030s, SSP5-8.5 | 1294.27 | 613.17 | 793.49 | 2700.93 | 1991.83 |
| 2050s, SSP1-2.6 | 1285.91 | 607.85 | 799.46 | 2693.22 | 1982.18 |
| 2050s, SSP2-4.5 | 1296.06 | 610.98 | 798.35 | 2705.39 | 1991.56 |
| 2050s, SSP5-8.5 | 1308.61 | 640.89 | 765.43 | 2714.93 | 1984.66 |

**Supplementary Figures**


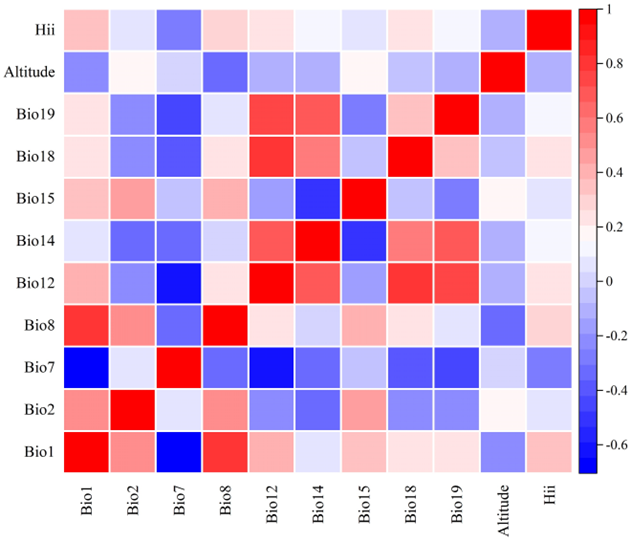


Figure S1. Correlation coefficients of environmental variables involved in model construction.


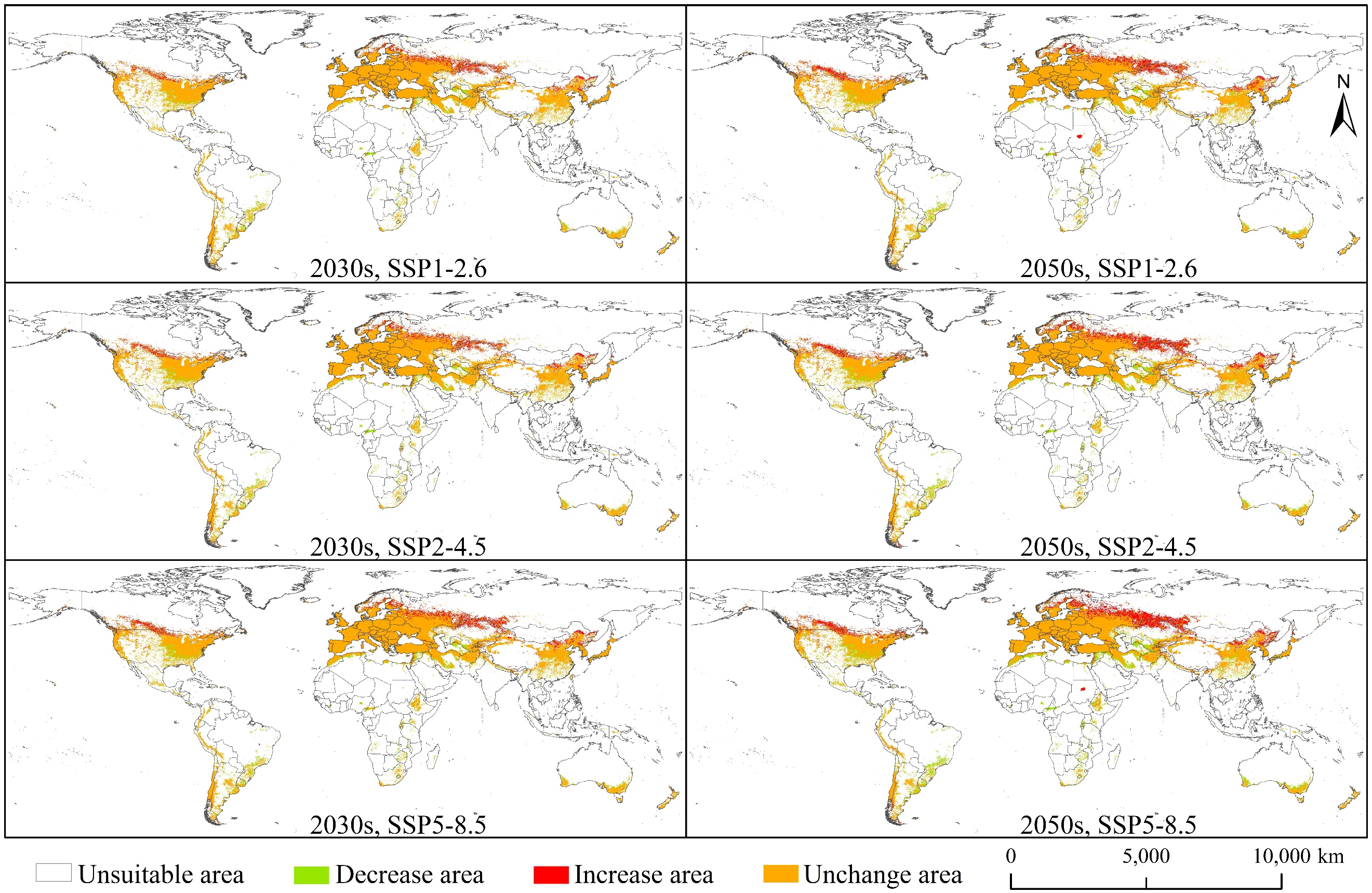


Figure S2 The changes to the potential suitable areas for *Erwinia amylovora* under future climatic conditions compared to near current climatic conditions.


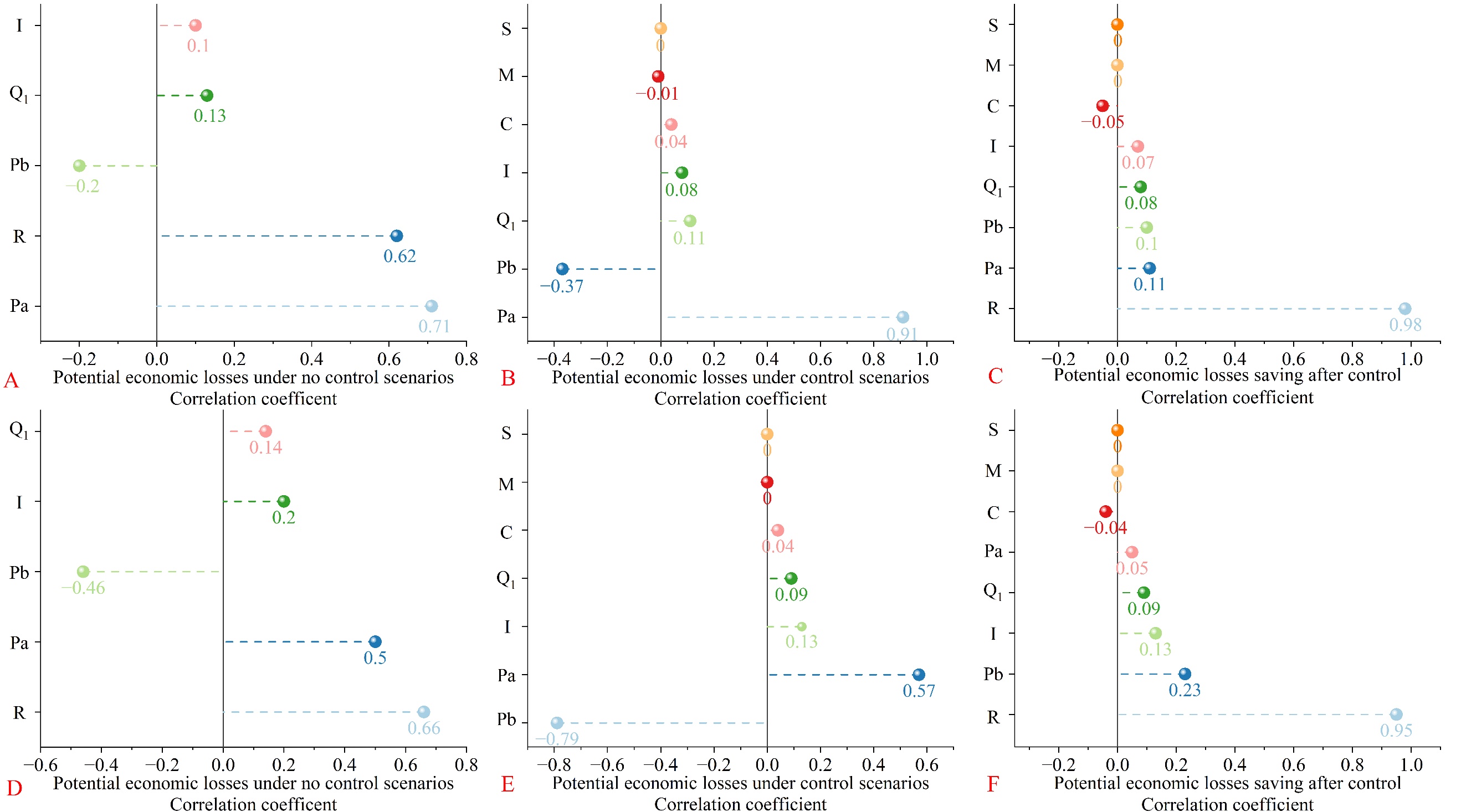


Figure S3. Sensitivity analysis results on potential economic losses to the pear (the A, B, and C) and apple (the D, E, and F) industry in China caused by *Erwinia amylovora* under the no-control scenario, control scenario, and saving after control.
